# Does the Supplementary Motor Area Keep Patients with Ondine's Curse Syndrome Breathing While Awake?

**DOI:** 10.1371/journal.pone.0084534

**Published:** 2014-01-24

**Authors:** Lysandre Tremoureux, Mathieu Raux, Anna L. Hudson, Anja Ranohavimparany, Christian Straus, Thomas Similowski

**Affiliations:** 1 UMR_S1158, Inserm-Université Paris 6, Paris, France; 2 Département d'Anesthésie Réanimation—Groupe Hospitalier Pitié-Salpêtrière Charles Foix, Assistance Publique—Hôpitaux de Paris, Paris, France; 3 Neuroscience Research Australia and University of New South Wales, Sydney, Australia; 4 Service Central des Explorations Fonctionelles Respiratoires de l'Exercice et de la Dyspnée—Groupe Hospitalier Pitié-Salpêtrière Charles Foix, Assistance Publique—Hôpitaux de Paris, Paris, France; 5 Centre National de Référence Maladies Rares pour le syndrome d'Ondine (adultes)—Groupe Hospitalier Pitié-Salpêtrière Charles Foix, Assistance Publique—Hôpitaux de Paris, Paris, France; 6 Service de Pneumologie et Réanimation Médicale—Groupe Hospitalier Pitié-Salpêtrière Charles Foix, Assistance Publique—Hôpitaux de Paris, Paris, France; University of Toronto, Canada

## Abstract

**Background:**

Congenital central hypoventilation syndrome (CCHS) is a rare neuro-respiratory disorder associated with mutations of the PHOX2B gene. Patients with this disease experience severe hypoventilation during sleep and are consequently ventilator-dependent. However, they breathe almost normally while awake, indicating the existence of cortical mechanisms compensating for the deficient brainstem generation of automatic breathing. Current evidence indicates that the supplementary motor area plays an important role in modulating ventilation in awake normal humans. We hypothesized that the wake-related maintenance of spontaneous breathing in patients with CCHS could involve supplementary motor area.

**Methods:**

We studied 7 CCHS patients (5 women; age: 20–30; BMI: 22.1±4 kg.m^−2^) during resting breathing and during exposure to carbon dioxide and inspiratory mechanical constraints. They were compared with 8 healthy individuals. Segments of electroencephalographic tracings were selected according to ventilatory flow signal, from 2.5 seconds to 1.5 seconds after the onset of inspiration. After artefact rejection, 80 or more such segments were ensemble averaged. A slow upward shift of the EEG signal starting between 2 and 0.5 s before inspiration (pre-inspiratory potential) was considered suggestive of supplementary motor area activation.

**Results:**

In the control group, pre-inspiratory potentials were generally absent during resting breathing and carbon dioxide stimulation, and consistently identified in the presence of inspiratory constraints (expected). In CCHS patients, pre-inspiratory potentials were systematically identified in all study conditions, including resting breathing. They were therefore significantly more frequent than in controls.

**Conclusions:**

This study provides a neurophysiological substrate to the wakefulness drive to breathe that is characteristic of CCHS and suggests that the supplementary motor area contributes to this phenomenon. Whether or not this “cortical breathing” can be taken advantage of therapeutically, or has clinical consequences (like competition with attentional resources) remains to be determined.

## Introduction

Congenital central hypoventilation syndrome (CCHS) is a rare neuro-respiratory disorder associated with several possible mutations of the PHOX2B gene [1]. Animal models have shown that these mutations result in major alterations in the development of the parafacial region of the brainstem that is instrumental to respiratory chemosensitivity [2]. Patients with these mutations experience hypoventilation when sleeping, resulting in sleep-related ventilator-dependency [1]. They lack ventilatory and perceptual responses to hypercapnia or hypoxia [1]. However, most CCHS patients have normal or subnormal resting ventilation when awake. The dramatic discrepancy between sleep and wake suggests the contribution of cortical mechanisms [3], but the precise nature of these mechanisms is currently unknown.

Ventilation of the lungs results from phasic contractions of respiratory muscles in response to a so-called “neural drive to breathe” that is relayed to the muscles by spinal respiratory motoneurons. The net output of spinal respiratory motoneurons depends on the various inputs that they receive not only from automatic brainstem central pattern generators but also from various suprapontine circuits that are responsible for emotional breathing modulations, voluntary breathing control and the interplay between respiration and nonrespiratory processes of cortical origin, primarily speech and voluntary movements [4], [5], [6]. Consequently, ventilatory behaviour at any given moment reflects the integration of voluntary and involuntary rhythmic and non-rhythmic central drives, further modulated by respiratory and nonrespiratory afferents [4].

In humans, the contribution of cortical processes to the overall drive to breathe is phenomenologically illustrated by certain particularities of breathing control. For example, decreasing carbon dioxide partial pressure in the blood by passive hyperventilation (ventilator-induced hypocapnia) is associated with apnoea during sleep, but rarely while awake [7], [8]. Similarly, when breathing difficulties are induced experimentally in sleeping individuals by inspiratory loading, ventilation tends to decrease [9]. In contrast, awake humans faced with such loads compensate or overcompensate for the inspiratory burden and tend to hyperventilate [10], [11]. Load compensation is accompanied by electroencephalographic (EEG) signs of activation of the supplementary motor area (SMA) in the form of slow EEG negativities recorded at the vertex that precede inspiration [12], [13]. These “pre-inspiratory potentials” (PIPs) resemble the premotor potentials that accompany movement preparation [Bereitschaftspotential] and are thought to originate in the SMA (Shibasaki, 2006). Transcranial magnetic stimulation studies have also established connections between the SMA and phrenic motoneurons [14], [15]. In addition, it has been shown that the SMA exerts a tonic facilitatory influence on phrenic motoneurones in humans, via the primary motor cortex [16].

Therefore, we hypothesized that resting ventilatory activity in awake CCHS patients would be associated with respiratory-related EEG signs of SMA activity, i.e. pre-inspiratory potentials. Such a respiratory-related cortical activity is normally not observed in healthy individuals.

## Methods

### Ethics statement

The study was conducted according to the principles expressed in the Declaration of Helsinki. Participants were informed about the general study procedure, the methods used and the absence of any associated risks, and gave their written consent to participate. The exact study objectives were only revealed to the subjects *post hoc* in order to limit bias. The study was approved by the appropriate local legal and ethics authority (Comité de Protection des Personnes Ile-de-France 6, Pitié-Salpêtrière, Paris).

### Patients and controls

#### Index group

CCHS patients with documented PHOX2B mutations, followed at the adult section of the French national CCHS reference centre, were systematically considered for inclusion. Exclusion criteria were: partial pressure of oxygen in arterial blood (PaO_2_) while breathing room air less than 65 mmHg; partial pressure of carbon dioxide in arterial blood (PaCO_2_) greater than 55 mmHg; daytime dependence on mechanical ventilation; significant restrictive or obstructive ventilatory defect on pulmonary function testing; use of any kind of neurotropic medication. According to these criteria, an index group of 7 patients was constituted (5 women and 2 men; age: 20–30 years, height: 172±7 cm, BMI: 22.1±4 kg.m^−2^). Of note, at the time of study, this index group represented the majority of the known French adult patients with CCHS. Two patients were tracheotomized. In one case, the tracheotomy orifice was blocked during the experiments and the patient breathed through the natural airway; in the other case, the measurements were performed with the patient breathing via the tracheotomy with the cuff inflated. One of the patients received oral contraception with desogestrel and was subsequently found to have recovered some degree of carbon dioxide chemosensitivity [17]. All patients were non-smokers.

#### Control group

The above index group was compared to a control group of healthy volunteers free of any cardiac, pulmonary or neuromuscular disorder (8 women, neither pregnant nor breastfeeding; age: 20–29 years; height: 164±3 cm, BMI: 21±1.4 kg.m^−2^). They were all non-smokers and totally naive to respiratory physiology experiments.

### Experimental conditions and protocol

The participants were asked to abstain from alcohol, avoid sleep deprivation and refrain from taking any psychotropic medication for 24 hours prior to the experimental sessions. Study participants were studied whilst seated comfortably in a chair that fully supported their neck, legs and arms. During the experimental sessions, they were distracted from their laboratory surroundings by listening to music of their choice. The investigators and recording equipment were placed out of their view.

The experimental protocol comprised 5 study conditions. “Control 1” (condition #1) consisted of resting ventilation with minimal constraints, during which the participants only wore a respiratory inductance plethysmography vest (see below). “Control 2” (condition #2) also consisted of resting ventilation, but the participants breathed through a respiratory measurement apparatus (see below). “Chemostimulation” (condition #3) involved breathing a gas mixture of either 5–7% CO_2_ in oxygen for 20 minutes —10 minutes stabilisation of the PetCO_2_ followed by 10 minutes of recording—. “Loading” (condition #4) involved breathing through an inspiratory threshold load with a 23 cmH_2_O load for 10 minutes. “Control 3” (condition #5) was identical to “Control 2”. Heart rate (HR) and transcutaneous pulse oximetry (SpO_2_) were monitored continuously as a safety measure.

### Respiratory measurements

#### Respiratory inductance plethysmography

Rib cage and abdomen displacements were recorded by two coils placed in a sleeveless jacket, only allowing circumferential stretching of the wires (Visuresp®, RBI, Meylan, France). The signal was digitised at 40 Hz and processed to provide a measurement of ventilatory flow (V'_IP_, for “inductance plethysmography”), as previously described [18].

#### Expired CO2

During “Control 2”, “Chemostimulation”, “Loading” and “Control 3”, the subjects wore a facial mask (Ultra Mirage®, ResMed Corp Poway, CA, USA) that gave access to expired air sampling for the measurement of end-tidal partial CO_2_ pressure (PetCO_2_). This was achieved with an infrared gas analyzer connected to the mask (CO_2_ pump flow 150 cm^3^.min^−1^, IR1505, Servomex, Plaine Saint Denis, France). PetCO_2_ was only used to monitor the stability of the hypercapnic stimulations.

### Electroencephalography

**Figure 1:**
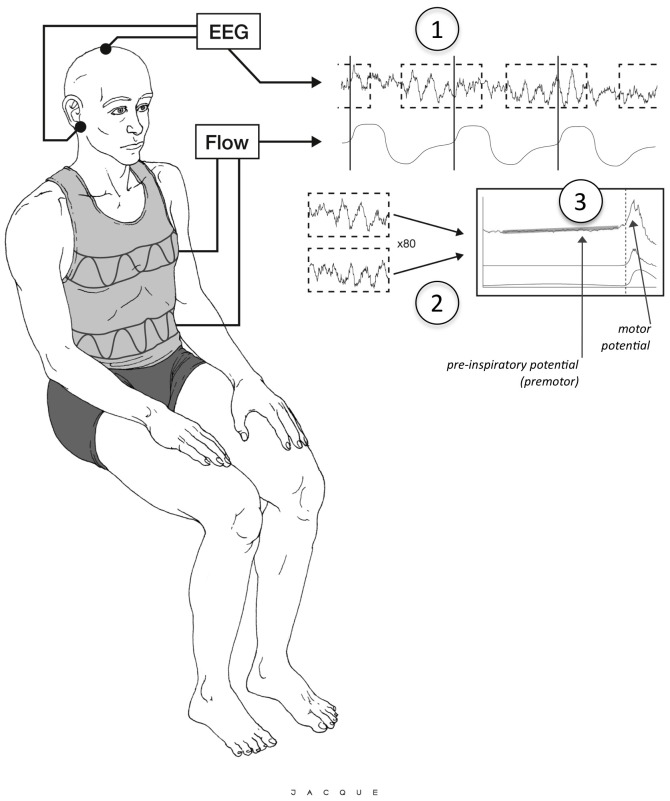
Schematic representation of the method used to identify pre-inspiratory potentials from the raw EEG signal and the ventilatory flow signal. (Adapted from Raux et al., *Anesthesiology*—[19]—with permission from the authors and the publisher.) Artwork Robin Jacqueline. The EEG signal is segmented in epochs defined according to the ventilatory flow signal (1). These epochs are ensemble averaged (2). The resulting signal is inspected visually for a putative pre-inspiratory potential (3) of which the presence is ascertained through the calculation of a linear regression over the region of interest and comparison of the slope of this regression with 0. See “Methods” for details. Pre-inspiratory potentials and the related motor potentials are normally absent during quiet breathing.

Surface scalp electroencephalographic signal (EEG) was recorded using a 12-electrode cap installed after rubbing and cleaning with alcohol and application of a conductive gel (EasyCap, Brain Products GmbH, Germany). Active electrodes were placed in equidistant positions (ActiCap, Brain Products GmbH, Germany) according to the conventional “10–20” topographic system. The earth electrode was positioned at AFz. The EEG signal was digitised at 2000 Hz and recorded using V-Amp® software (Brain Products GmbH, Germany) for subsequent processing, which was performed according to the method previously described [12], [19] (Figure 1. Offline, EEG signals were referenced to linked earlobe electrodes. In each of the study conditions, eighty 4-second EEG epochs (from 2.5 s before to 1.5 s after onset of inspiration defined as the point of zero flow) on the V'_IP_ signal were created. The EEG signal was filtered between 0.01 and 5 Hz. EEG epochs with clear artefacts (EEG gradient greater than 5 µV.ms^−1^; EEG amplitude greater than 50 µV) or EEG consistent with eye movements were discarded and the remaining epochs were ensemble averaged. Averaged tracings were examined for the presence of an inspiratory premotor activity in the form of a slow upward shift of the EEG signal starting between 2 and 0.5 s before inspiration. When this activity was observed, a first-order least-squares regression equation was fitted to the corresponding segment of EEG. A pre-inspiratory potential was considered to be present when and only when the slope of this equation was positive and significantly different from zero according to the F-test for equality of variance. The latency of the pre-inspiratory potential, if present, was defined as the interval in ms between the negativity and the start of inspiration. The amplitude of the pre-inspiratory potential corresponded to the potential measured in µV between baseline and the “zero flow” point.

### Statistical analysis

All statistics were performed using Prism 4® software (GraphPad Software, Inc., CA, USA). The normality of the distribution of the results was tested using a Shapiro-Wilk test. As the hypothesis of normality was not verified for all variables, continuous variables are expressed as their median and 95% confidence intervals (CI_95_). Between group comparisons of dichotomous variables (presence or absence of a PIP) were performed using Fisher's exact test. Latencies and amplitudes were compared between groups only in the “Load” condition in which all subjects of both groups exhibited a PIP (see below, results). This comparison was performed using a Mann-Whitney test. Differences were considered significant when the probability *P* of a type I error was less than 5%.

## Results

### Control group

**Figure 2:**
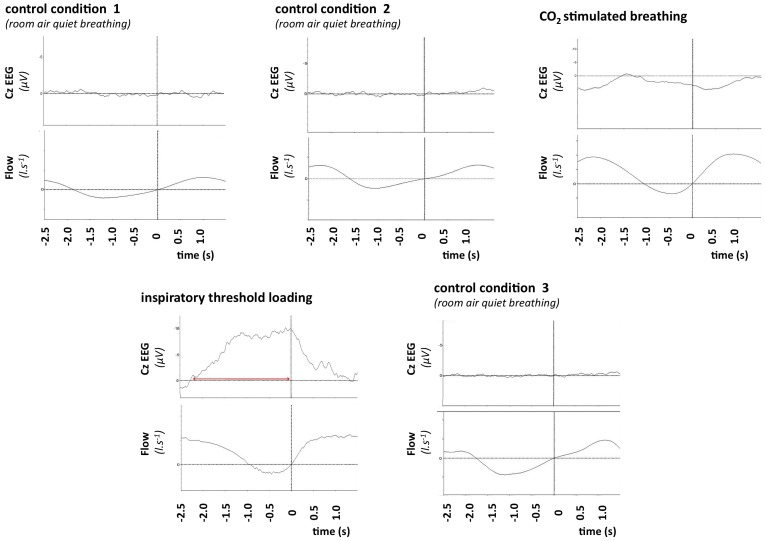
Average pre-inspiratory EEG tracings in one of the control subjects. In each of the panels, the top trace depicts the Cz-EEG signal, and the bottom trace depicts ventilatory flow. The vertical line indicates the onset of inspiration. In the three “control condition” panels (control 1: resting ventilation with minimal constraint, namely a respiratory inductance plethysmography vest only; control 2: resting ventilation while breathing through a pneumoatchograph; control 3: as control 2, but during the washout period following inspiratory loading), inspiration is not preceded by any change in the EEG signal (absence of pre-inspiratory potentials). In the “CO_2_ stimulated breathing” panel, inspiration is also not preceded by any change in the EEG signal (absence of pre-inspiratory potentials). In contrast, in the “inspiratory threshold loading” panel, inspiration is preceded by a shift upward of the EEG trace (horizontal double arrowed red line) that is characteristic of a pre-inspiratory potential. This pattern exactly corresponds to what is expected in normal individuals [12].

All subjects in the control group participated in all sessions. PIPs were inconsistent during spontaneous breathing (“Control 1”, “Control 2”, “Control 3”) and during “Chemostimulation”, as one subject exhibited a PIP during “Control 1”, 3 during “Control 2”, 1 during “Control 3”. PIPs were also inconsistent during “Chemostimulation” (2 out of 8 subjects). In contrast, PIPs were systematically present during “Loading” (8 out of 8 subjects). PIPs were clearly visible in the Cz derivation and none of the other ones. Figure 2 provides an example of the EEG tracings obtained in one subject in the various conditions.

### Patients

**Figure 3:**
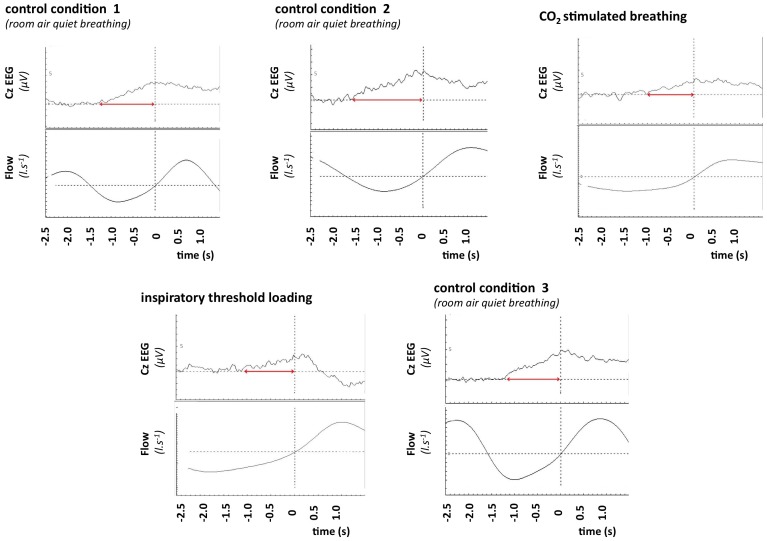
Average pre-inspiratory EEG tracings in one of the congenital central hypoventilation syndrome patient. In each of the panels, the top trace depicts the Cz-EEG signal, and the bottom trace depicts ventilatory flow. The vertical line indicates the onset of inspiration. In the “inspiratory threshold loading” panel, inspiration is preceded by a shift upward of the EEG trace (horizontal double arrowed red line) that is characteristic of a pre-inspiratory potential. This observation is similar to that made in normal individuals [12]. In contrast to normal individuals however, a pre-inspiratory potential can also be seen, abnormally, in the three “control condition” panels (control 1: resting ventilation with minimal constraint, namely a respiratory inductance plethysmography vest only; control 2: resting ventilation while breathing through a pneumoatchograph; control 3: as control 2, but during the washout period following inspiratory loading) and in the “CO_2_ stimulated breathing” panel.

Two patients dropped out of the study following “Chemostimulation” due to CO_2_-induced headache. One patient only participated in “Control 1”, “Chemostimulation” and “Load”. All patients exhibited PIPs in all study conditions in which they participated. PIPs were clearly visible in the Cz derivation and none of the other ones. Figure 3 provides an example of the tracings obtained in one subject in the various conditions.

Consequently, PIPs were significantly more frequent in patients than healthy control subjects during all control conditions (“Control 1”, *P* = 0.001; “Control 2”, *P* = 0.031; “Control 3”, *P* = 0.01) and during “Chemostimulation” (*P* = 0.006). No significant difference was observed during “Loading” (*P* = 1.000).

During “Loading”, the median latency of PIPs was 1.8 s [1.1–2.0] in patients vs. 1.6 s [1.4–2.2] in controls (no statistically significant difference). The corresponding values for median amplitude were 5.9 µV [2.1–14] and 3.7 µV [2.1–8.9], respectively (no statistically significant difference). Of note, although statistical comparison of amplitudes and latencies was not possible in the other conditions due to the very small sample sizes, the amplitudes of PIPs inconsistently observed in controls were always much lower (5- to 10-fold) than those of the PIPs observed in patients. PIP latencies were also much shorter (2- to 4-fold) in controls.

## Discussion

This study shows that awake, spontaneously breathing adult CCHS patients exhibit a respiratory-related cortical activity that is normally absent during resting ventilation in healthy subjects.

### Methodological considerations

Available data predict that PIPs should generally not be detected during resting breathing, not be detected during exercise and not be detected during CO_2_-stimulated breathing in normal individuals; they also predict that PIPs should consistently be present in response to inspiratory threshold loading [12], [13], [19], [20], [21]. This was mostly the case in our control subjects, but “unexpected” PIPs were observed. Similar observations have been made in previous studies conducted by our group (one subject out of ten in [12]; one subject out of nine in [13]; two subjects out of seven in [19]). In this study as in the previous ones, and despite the various precautions taken (see methods), we cannot exclude the possibility that some subjects focused on their respiratory activity, as suggested by the higher rate of PIPs during “Control 2” (breathing through a face mask) than during “Control 1” (minimally constrained breathing, with only a respiratory inductive plethysmography vest). This could also account for the higher rate of resting breathing PIPs during “Control 3”, i.e. at a point of the experimental protocol at which the subjects would have realized that their breathing was being manipulated. It is also possible that some CCHS patients also had a PIP due to the experimental setting rather than to physiological reasons. Yet the comparison of our CCHS group with a control group of similar size was sufficient to evidence a statistically significant difference in the incidence of PIPs during spontaneous breathing and during CO_2_-stimulated breathing. This incidence was higher in the CCHS patients than in the normal individuals, and the levels of significance of the control-CCHS differences were at their highest in the two conditions where the comparison was the most important, namely the “control 1” condition (room air quiet breathing with minimal respiratory apparatus, p = 0.001) and the “CO_2_ stimulated breathing” condition (p = 0.006). When PIPs were present in the normal subjects, they were several folds smaller than in the CCHS subjects. We therefore do not think that the small size of the control group (and the fact that the two groups were not exactly gender matched) compromises the physiological validity of our observations.

### Neurophysiological considerations

#### SMA activity and “cortical drive to breathe” in CCHS

Studies using transcranial magnetic stimulation have demonstrated a direct connection between the SMA and phrenic motoneurones [15] and that the SMA can modulate the pathway from the primary motor cortex to the phrenic motoneurones in both an inhibitory and an excitatory manner [16], [21]. These studies suggest that the SMA is implicated in the cortical control of breathing in healthy subjects. Moreover, EEG and functional imaging studies have demonstrated SMA activation during voluntary inspirations [22], [23] and during the non-volitional response to inspiratory constraints [12], [24]. Therefore, the results of the present study suggest the SMA is involved in wake-related maintenance of rhythmic breathing in CCHS. However, the nature of this involvement has yet to be elucidated. Breath-by-breath “cortical control” of breathing could be postulated, [6], [25], but its neurophysiological basis remains to be established (see below, “cortical automatization and interferences”). Alternatively, a “cooperative” mechanism involving facilitation at the level of spinal respiratory motoneurons could be proposed, as follows. Spinal respiratory motoneurons integrate various voluntary and involuntary rhythmic and non-rhythmic respiratory central drives [4], [5], [26]. These descending inputs interfere with one another: how spinal respiratory motoneurons react to a given input depends on how other prior inputs have modified their membrane polarity. For example, tidal inspiration during eucapnic resting breathing facilitates the diaphragm response to transcranial magnetic stimulation [27]. Increasing the bulbospinal ventilatory drive by CO_2_ stimulation also strongly facilitates the response of the diaphragm to corticospinal inputs generated by transcranial magnetic stimulation [28], [29]. The site of this facilitation appears to be spinal [29], [30]. Also, that the inspiratory output from parasternal intercostal motoneurones is altered by voluntary trunk rotation (presumably due to corticospinal projections that decrease motoneurone threshold) [31], but that the same postural contraction has no effect on phrenic inspiratory motoneurone output [32] provides further evidence of the integration of different descending drives at the respiratory interneurones and motoneurones at the spinal cord. It could be postulated that, in awake CCHS patients, the SMA-phrenic input described in this study may be sufficient to make phrenic motoneurons responsive to the “residual” automatic ventilatory command present in many of these patients. According to this hypothesis, the loss of intracortical connectivity characteristic of sleep [33] would contribute to sleep-related hypoventilation in CCHS. Fragmentary observations from our group suggest that some CCHS patients exhibit abnormally rapid diaphragm responses to TMS during relaxation (unpublished data), which would be consistent with a “facilitatory tone”. Of note, the fact that CCHS patients with certain Phox2B mutations have no residual ventilatory activity even awake is an argument against the hypothesis of a cortical “ectorhythm” [6] and in favour of the spinal facilitation mechanism described above.

#### Cortical automatization and interferences

A respiratory-related motor cortical activity does not necessarily mean that “each breath is taken voluntarily”. There are numerous examples of automatized voluntary motor actions that can be performed “without attention being clearly directed toward the details of the movement” [34]. Another characteristic of the automatization of a learned movement is that “performance does not deteriorate if another task is performed simultaneously” [35]. Breathing while awake in CCHS patients has been purported to satisfy these two criteria by Shea et al. [36] who reported that mental activities, such as reading, arithmetic, or video gaming, were not associated with hypoventilation in children with CCHS. However, conflicting observations have been reported [37]. They may be difficult to interpret because healthy children can also exhibit decreased ventilatory activity in relation to attentional load and its emotional content [38]. Of note, the SMA belongs to cortical structures believed to be involved in automatization processes [34]: data from our group suggests that respiratory automatization rapidly occurs during inspiratory loading, with a strong SMA involvement [24].

Irrespective of the previous observations, all made in children, our findings raise the question of putative “competition” for cortical resources in adult CCHS patients. In other words, can their respiratory-related cortical activity have an impact on their motor and/or cognitive performances? Voluntary breathing control disturbs simple motor tasks [39], [40], but whether or not this remains the case when voluntary breathing becomes automatic has not been clearly elucidated. Patients with chronic obstructive pulmonary disease (COPD), who by nature permanently fight an abnormal respiratory load, have normal manual tracking performances [41]. It could even be hypothesized that a sustained respiratory-related cortical activity could be beneficial on certain functions, because voluntary breathing enhances the response of nonrespiratory muscles to corticospinal inputs [42]: this could facilitate the execution of motor tasks. Of interest, some adult CCHS patients followed at our reference center anecdotally report that they concentrate more aptly on demanding intellectual tasks when under mechanical ventilation than when spontaneously breathing (one patient reported being used to put herself on her ventilator during academic exams). Specific studies will therefore need to determine whether or not the cortically driven breathing characteristic of awake CCHS patients negatively impacts neuromotor or cognitive performances.

## Conclusion

In conclusion, we have evidenced that pre-inspiratory potentials are present during resting ventilatory activity in awake CCHS patients, which suggests that SMA activation is required for maintenance of breathing in this setting. It is likely that this activation facilitates the response of spinal motoneurones to the residual bulbospinal drive to breathe. Further studies are needed to fully characterize the mechanisms of “cortical breathing” in CCHS, and to determine the clinical implications of these findings. Whether or not inducing spinal facilitatory plasticity through pharmacological or nonpharmacological interventions could have therapeutic benefits will also have to be evaluated.
